# Penta­kis(2-oxo-2,3-dihydro­pyrimidin-1-ium) di-μ_3_-chlorido-tri-μ_2_-chlorido-hexa­chloridotricadmate(II)

**DOI:** 10.1107/S160053680801862X

**Published:** 2008-06-25

**Authors:** Mukhtar A. Kurawa, Christopher J. Adams, A. Guy Orpen

**Affiliations:** a School of Chemistry, University of Bristol, Bristol BS8 1TS, England

## Abstract

The title compound, (C_4_H_5_N_2_O)_5_[Cd_3_Cl_11_], was obtained from the reaction of 2-hydroxy­pyrimidine hydro­chloride and cadmium(II) chloride in concentrated HCl solution. The crystal structure consists of planar 2-oxo-1,2-dihydro­pyrimidin-3-ium cations with both N atoms protonated and the O atom unprotonated, and a complex trinuclear [Cd_3_Cl_11_]^5−^ anion of approximately *D*_3*h*_ symmetry, which has a triangle of three octa­hedrally coordinated Cd^II^ centres bonded to 11 chloride ions. Three of the chloride ions bridge adjacent Cd atoms, two cap the faces of the Cd_3_ triangle and the remaining six are terminally bonded and act as hydrogen-bond acceptors. Various N—H⋯Cl hydrogen bonds connect the anions and cations and, in addition, inter­molecular N—H⋯O hydrogen bonds contribute to the formation of a three-dimensional network.

## Related literature

A related salt of the same anion in the ortho­rhom­bic crystal system has been reported with [(CH_3_)_2_NH_2_]^+^ cations (Waśkowska *et al.*, 1990 ▶), while Furberg & Aas (1975 ▶) described the structure of the same cation as its chloride salt.
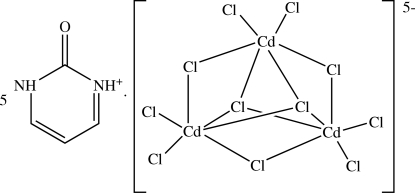


## Experimental

### 

#### Crystal data

(C_4_H_5_N_2_O)_5_[Cd_3_Cl_11_]
*M* *_r_* = 1212.65
Monoclinic, 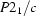

*a* = 17.5446 (2) Å
*b* = 8.08980 (2) Å
*c* = 27.2451 (6) Å
β = 104.9470 (2)°
*V* = 3736.12 (10) Å^3^
*Z* = 4
Mo *K*α radiation
μ = 2.53 mm^−1^
*T* = 100 (2) K
0.51 × 0.07 × 0.04 mm

#### Data collection

Oxford Diffraction Gemini-R Ultra diffractometer
Absorption correction: multi-scan (*CrysAlis RED*; Oxford Diffraction, 2007 ▶) *T*_min_ = 0.522, *T*_max_ = 0.91
65985 measured reflections
10979 independent reflections
7919 reflections with *I* > 2σ(*I*)
*R*_int_ = 0.046

#### Refinement

*R*[*F* ^2^ > 2σ(*F* ^2^)] = 0.025
*wR*(*F* ^2^) = 0.058
*S* = 0.96
10979 reflections
442 parameters
H-atom parameters constrained
Δρ_max_ = 0.99 e Å^−3^
Δρ_min_ = −1.09 e Å^−3^

### 

Data collection: *CrysAlis CCD* (Oxford Diffraction, 2007 ▶); cell refinement: *CrysAlis RED* (Oxford Diffraction, 2007 ▶); data reduction: *CrysAlis RED*; program(s) used to solve structure: *SHELXS97* (Sheldrick, 2008 ▶); program(s) used to refine structure: *SHELXL97* (Sheldrick, 2008 ▶); molecular graphics: *SHELXTL* (Sheldrick, 2008 ▶); software used to prepare material for publication: *SHELXTL*.

## Supplementary Material

Crystal structure: contains datablocks I, global. DOI: 10.1107/S160053680801862X/lh2641sup1.cif

Structure factors: contains datablocks I. DOI: 10.1107/S160053680801862X/lh2641Isup2.hkl

Additional supplementary materials:  crystallographic information; 3D view; checkCIF report

## Figures and Tables

**Table 1:** Selected bond lengths (Å)

Cd1—Cl2	2.5216 (6)
Cd1—Cl1	2.5343 (6)
Cd1—Cl5	2.6796 (6)
Cd1—Cl3	2.6900 (6)
Cd1—Cl4	2.6917 (6)
Cd1—Cl6	2.7670 (6)
Cd2—Cl10	2.5184 (6)
Cd2—Cl11	2.5273 (6)
Cd2—Cl6	2.6698 (6)
Cd2—Cl3	2.6766 (6)
Cd2—Cl9	2.7295 (6)
Cd2—Cl4	2.7468 (6)
Cd3—Cl8	2.5081 (6)
Cd3—Cl7	2.5444 (6)
Cd3—Cl5	2.6284 (6)
Cd3—Cl9	2.6692 (6)
Cd3—Cl4	2.7201 (6)
Cd3—Cl6	2.7214 (6)

**Table 2:** Hydrogen-bond geometry (Å, °)

*D*—H⋯*A*	*D*—H	H⋯*A*	*D*⋯*A*	*D*—H⋯*A*
N2—H2*A*⋯Cl11^i^	0.86	2.56	3.246 (2)	138
N3—H3*A*⋯Cl8^ii^	0.86	2.37	3.104 (2)	144
N4—H4*A*⋯Cl10^iii^	0.86	2.31	3.160 (2)	169
N5—H5*A*⋯Cl7^iv^	0.86	2.41	3.194 (2)	151
N6—H6*A*⋯Cl1^v^	0.86	2.31	3.138 (2)	162
N7—H7*A*⋯O2^vi^	0.86	2.03	2.880 (3)	167
N10—H10*B*⋯Cl7^vi^	0.86	2.54	3.349 (2)	157
N8—H8*A*⋯O5^vii^	0.86	2.28	2.804 (3)	120
N8—H8*A*⋯O4^viii^	0.86	2.34	3.117 (4)	150
N9—H9*B*⋯O3	0.86	2.13	2.920 (3)	152

Symmetry codes: (i) 
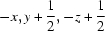
; (ii) 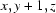; (iii) 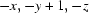; (iv) 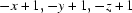; (v) 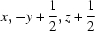; (vi) 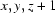; (vii) 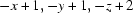; (viii) 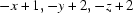.

**Fig. 1.**
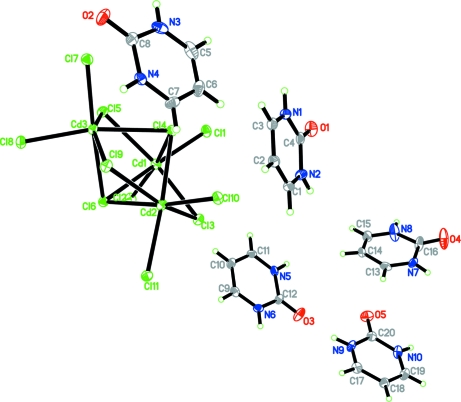
The molecular structure of I with atom labels and 50% probability displacement ellipsoids for non-H atoms.

**Fig. 2.**
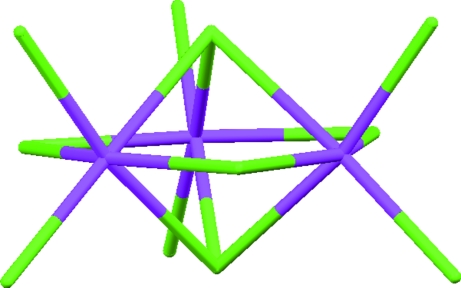
Geometry of the [Cd3Cl11]5- anion.

**Fig. 3.**
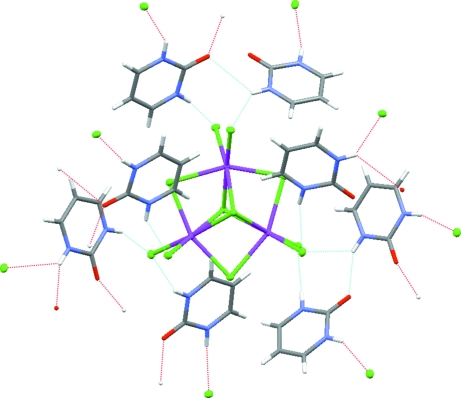
Hydrogen bond (dotted lines) environment around the [Cd3Cl11]5-anion.

## supplementary crystallographic information

### Comment

We sought to widen the use and exploitation of N—H···Cl interactions in the preparation of crystalline metal complexes by preparing [CdCl_4_][C_4_H_5_N_2_O]_2_ and the coordination network [CdCl_2_(C_4_H_4_N_2_O)_2_]. However, the title compound I was obtained instead, crystallizing in a monoclinic cell with the *P*2_1_/*c* space group and an asymmetric unit consisting of five [C_4_H_5_N_2_O]^+^ cations and one [Cd_3_Cl_11_]^5-^ anion. The crystal structure of a related complex determined at room temperature with a [Cd_3_Cl_11_]^5-^anion and [(CH_3_)_2_NH_2_]^+^ cations in the *Cmcm* space group revealed two alternating layers of cations and anions parallel to the (0 0 1) plane. This arrangement differs from that in the title compound I due to a complex three-dimensional hydrogen bond network involving anion-cation N—H···Cl and cation-cation N—H···O bonds. In contrast, the pyrimidin-2-onium cations reported in the related crystal structure (Furberg & Aas, 1975) display no N—H···O interactions.

### Experimental

The title compound was obtained from an attempt to synthesize bis-2-hydroxypyrimidinium tetrachlorocadmate(II). 2-hydroxypyrimidine hydrochloride and cadmium(II) chloride in a 2:1 molar ratio were dissolved in concentrated hydrochloric acid solution. This was left to evaporate slowly at room temperature and resulted in the formation of needle-shaped colourless crystals.

### Refinement

H atoms were positioned geometrically and refined using a riding model, with C—H = 0.93 Å and N—H = 0.86 Å and *U*_iso_(H) = 1.2 times *U*_eq_(C, N).

### Crystal data

**Table:**

C_4_H_5_N_2_O)_5_[Cd_3_Cl_11_]	*F*_000_ = 2344
*M**_r_* = 1212.65	*D*_x_ = 2.156 Mg m^−^^3^
Monoclinic, *P*2_1_/*c*	Mo *K*α radiation λ = 0.71073 Å
Hall symbol: -P 2ybc	Cell parameters from 28683 reflections
*a* = 17.5446 (2) Å	θ = 2.3–30.0º
*b* = 8.08980 (2) Å	µ = 2.53 mm^−^^1^
*c* = 27.2451 (6) Å	*T* = 100 (2) K
β = 104.9470 (2)º	Needle, colourless
*V* = 3736.12 (10) Å^3^	0.51 × 0.07 × 0.04 mm
*Z* = 4	

### Data collection

**Table:**

Oxford Diffraction Gemini-R Ultra diffractometer	10979 independent reflections
Radiation source: fine-focus sealed tube	7919 reflections with *I* > 2σ(*I*)
Monochromator: graphite	*R*_int_ = 0.046
Detector resolution: 10.4752 pixels mm^-1^	θ_max_ = 30.1º
*T* = 100(2) K	θ_min_ = 2.4º
1° width ω scans	*h* = −15→24
Absorption correction: multi-scan(CrysAlis RED; Oxford Diffraction, 2007)	*k* = −11→11
*T*_min_ = 0.522, *T*_max_ = 0.91	*l* = −38→38
65985 measured reflections	

### Refinement

**Table:**

Refinement on *F*^2^	Secondary atom site location: difference Fourier map
Least-squares matrix: full	Hydrogen site location: inferred from neighbouring sites
*R*[*F*^2^ > 2σ(*F*^2^)] = 0.025	H-atom parameters constrained
*wR*(*F*^2^) = 0.058	*w* = 1/[σ^2^(*F*_o_^2^) + (0.0307*P*)^2^] where *P* = (*F*_o_^2^ + 2*F*_c_^2^)/3
*S* = 0.96	(Δ/σ)_max_ = 0.001
10979 reflections	Δρ_max_ = 0.99 e Å^−^^3^
442 parameters	Δρ_min_ = −1.09 e Å^−^^3^
Primary atom site location: structure-invariant direct methods	Extinction correction: none

### Special details

**Table:**

Geometry. All e.s.d.'s (except the e.s.d. in the dihedral angle between two l.s. planes) are estimated using the full covariance matrix. The cell e.s.d.'s are taken into account individually in the estimation of e.s.d.'s in distances, angles and torsion angles; correlations between e.s.d.'s in cell parameters are only used when they are defined by crystal symmetry. An approximate (isotropic) treatment of cell e.s.d.'s is used for estimating e.s.d.'s involving l.s. planes.
Refinement. Refinement of *F*^2^ against ALL reflections. The weighted *R*-factor *wR* and goodness of fit *S* are based on *F*^2^, conventional *R*-factors *R* are based on *F*, with *F* set to zero for negative *F*^2^. The threshold expression of *F*^2^ > σ(*F*^2^) is used only for calculating *R*-factors(gt) *etc*. and is not relevant to the choice of reflections for refinement. *R*-factors based on *F*^2^ are statistically about twice as large as those based on *F*, and *R*- factors based on ALL data will be even larger.

### Fractional atomic coordinates and isotropic or equivalent isotropic displacement parameters (Å^2^)

**Table:**

	*x*	*y*	*z*	*U*_iso_*/*U*_eq_	
Cd1	0.278566 (10)	0.40453 (2)	0.234610 (6)	0.01260 (4)	
Cd2	0.096112 (10)	0.34002 (2)	0.139514 (6)	0.01313 (4)	
Cd3	0.278973 (10)	0.37558 (2)	0.104602 (6)	0.01268 (4)	
Cl1	0.31283 (3)	0.64928 (8)	0.29430 (2)	0.01631 (13)	
Cl2	0.34187 (4)	0.18017 (8)	0.29589 (2)	0.01652 (13)	
Cl3	0.12927 (3)	0.36223 (8)	0.24084 (2)	0.01473 (12)	
Cl4	0.20555 (3)	0.59016 (7)	0.15423 (2)	0.01321 (12)	
Cl5	0.39418 (3)	0.42065 (8)	0.18691 (2)	0.01426 (12)	
Cl6	0.22670 (3)	0.15581 (7)	0.16419 (2)	0.01246 (11)	
Cl7	0.31718 (4)	0.59045 (8)	0.04703 (2)	0.01607 (12)	
Cl8	0.33408 (3)	0.13362 (8)	0.06752 (2)	0.01601 (12)	
Cl9	0.13161 (3)	0.33560 (8)	0.04770 (2)	0.01508 (12)	
Cl10	−0.01624 (3)	0.54066 (8)	0.11041 (2)	0.01515 (12)	
Cl11	0.00531 (3)	0.09258 (8)	0.12829 (2)	0.01516 (12)	
N1	0.17631 (12)	0.9564 (3)	0.27179 (8)	0.0163 (4)	
H1A	0.2120	1.0282	0.2707	0.020*	
N2	0.10684 (12)	0.8001 (3)	0.31601 (8)	0.0177 (5)	
H2A	0.0971	0.7685	0.3439	0.021*	
N3	0.19690 (13)	0.9043 (3)	0.01223 (8)	0.0202 (5)	
H3A	0.2383	0.9631	0.0138	0.024*	
N4	0.10513 (11)	0.7144 (3)	−0.02736 (7)	0.0140 (4)	
H4A	0.0860	0.6475	−0.0520	0.017*	
N5	0.57141 (12)	0.1754 (3)	0.87043 (8)	0.0174 (5)	
H5A	0.5851	0.2418	0.8958	0.021*	
N6	0.48648 (12)	−0.0147 (3)	0.82295 (7)	0.0172 (5)	
H6A	0.4442	−0.0732	0.8171	0.021*	
N7	0.35772 (12)	0.7374 (3)	0.92554 (8)	0.0194 (5)	
H7A	0.3084	0.7532	0.9232	0.023*	
N8	0.48815 (14)	0.7805 (3)	0.96474 (10)	0.0385 (7)	
H8A	0.5249	0.8278	0.9875	0.046*	
N9	0.30200 (12)	0.1727 (3)	0.89707 (8)	0.0199 (5)	
H9B	0.3412	0.1411	0.8859	0.024*	
N10	0.25130 (13)	0.3093 (3)	0.95573 (8)	0.0214 (5)	
H10B	0.2572	0.3686	0.9827	0.026*	
O1	0.20540 (11)	0.9741 (2)	0.35816 (6)	0.0235 (4)	
O2	0.20021 (11)	0.7968 (3)	−0.06516 (7)	0.0250 (4)	
O3	0.46339 (11)	0.0904 (3)	0.89546 (7)	0.0282 (5)	
O4	0.39372 (14)	0.9435 (3)	0.98438 (8)	0.0467 (7)	
O5	0.38187 (11)	0.3297 (3)	0.95908 (7)	0.0270 (5)	
C1	0.06351 (14)	0.7355 (3)	0.27248 (9)	0.0174 (5)	
H1B	0.0246	0.6583	0.2734	0.021*	
C2	0.07546 (15)	0.7811 (3)	0.22687 (9)	0.0172 (5)	
H2B	0.0450	0.7379	0.1965	0.021*	
C3	0.13409 (15)	0.8928 (3)	0.22769 (9)	0.0173 (5)	
H3B	0.1448	0.9251	0.1974	0.021*	
C4	0.16646 (14)	0.9148 (3)	0.31878 (9)	0.0166 (5)	
C5	0.16286 (17)	0.9166 (3)	0.05050 (10)	0.0244 (6)	
H5B	0.1841	0.9873	0.0775	0.029*	
C6	0.09702 (16)	0.8263 (3)	0.05048 (10)	0.0223 (6)	
H6B	0.0723	0.8347	0.0767	0.027*	
C7	0.06914 (15)	0.7229 (3)	0.01003 (9)	0.0183 (6)	
H7B	0.0248	0.6582	0.0087	0.022*	
C8	0.17070 (14)	0.8045 (3)	−0.02962 (9)	0.0158 (5)	
C9	0.53129 (14)	−0.0260 (3)	0.79005 (9)	0.0178 (5)	
H9A	0.5166	−0.0976	0.7625	0.021*	
C10	0.59844 (15)	0.0668 (3)	0.79652 (10)	0.0193 (6)	
H10A	0.6296	0.0616	0.7736	0.023*	
C11	0.61754 (15)	0.1676 (3)	0.83830 (10)	0.0186 (6)	
H11A	0.6631	0.2314	0.8444	0.022*	
C12	0.50373 (14)	0.0846 (3)	0.86560 (9)	0.0177 (5)	
C13	0.37700 (16)	0.6242 (3)	0.89528 (10)	0.0208 (6)	
H13A	0.3375	0.5698	0.8714	0.025*	
C14	0.45477 (15)	0.5872 (3)	0.89906 (10)	0.0194 (6)	
H14A	0.4693	0.5128	0.8771	0.023*	
C15	0.50936 (16)	0.6647 (4)	0.93653 (11)	0.0263 (6)	
H15A	0.5623	0.6360	0.9424	0.032*	
C16	0.41168 (17)	0.8310 (4)	0.96040 (10)	0.0271 (7)	
C17	0.23033 (16)	0.1194 (3)	0.87278 (10)	0.0205 (6)	
H17A	0.2239	0.0543	0.8439	0.025*	
C18	0.16560 (15)	0.1604 (3)	0.89027 (10)	0.0202 (6)	
H18A	0.1152	0.1243	0.8738	0.024*	
C19	0.17895 (16)	0.2565 (4)	0.93296 (9)	0.0222 (6)	
H19A	0.1370	0.2852	0.9463	0.027*	
C20	0.31738 (16)	0.2755 (3)	0.93906 (9)	0.0181 (6)	

### Atomic displacement parameters (Å^2^)

**Table:**

	*U*^11^	*U*^22^	*U*^33^	*U*^12^	*U*^13^	*U*^23^
Cd1	0.01208 (9)	0.01435 (10)	0.01056 (8)	−0.00011 (7)	0.00147 (6)	−0.00025 (7)
Cd2	0.00896 (8)	0.01588 (10)	0.01383 (8)	0.00031 (7)	0.00164 (6)	0.00023 (7)
Cd3	0.01154 (8)	0.01546 (10)	0.01122 (8)	0.00016 (7)	0.00326 (6)	−0.00117 (7)
Cl1	0.0137 (3)	0.0161 (3)	0.0176 (3)	0.0018 (2)	0.0013 (2)	−0.0036 (2)
Cl2	0.0193 (3)	0.0164 (3)	0.0130 (3)	0.0026 (3)	0.0026 (2)	0.0012 (2)
Cl3	0.0128 (3)	0.0178 (3)	0.0141 (3)	0.0005 (2)	0.0043 (2)	−0.0001 (2)
Cl4	0.0125 (3)	0.0129 (3)	0.0139 (3)	0.0005 (2)	0.0026 (2)	0.0000 (2)
Cl5	0.0106 (3)	0.0175 (3)	0.0140 (3)	−0.0005 (2)	0.0020 (2)	−0.0003 (2)
Cl6	0.0109 (3)	0.0132 (3)	0.0132 (3)	0.0000 (2)	0.0029 (2)	0.0001 (2)
Cl7	0.0183 (3)	0.0148 (3)	0.0151 (3)	0.0004 (2)	0.0044 (2)	0.0003 (2)
Cl8	0.0148 (3)	0.0164 (3)	0.0171 (3)	0.0018 (2)	0.0044 (2)	−0.0028 (2)
Cl9	0.0128 (3)	0.0192 (3)	0.0122 (3)	0.0010 (2)	0.0014 (2)	−0.0006 (2)
Cl10	0.0121 (3)	0.0149 (3)	0.0167 (3)	0.0013 (2)	0.0004 (2)	−0.0023 (2)
Cl11	0.0124 (3)	0.0158 (3)	0.0174 (3)	−0.0011 (2)	0.0041 (2)	−0.0009 (2)
N1	0.0155 (11)	0.0148 (12)	0.0193 (10)	0.0001 (9)	0.0058 (8)	0.0014 (9)
N2	0.0215 (11)	0.0188 (12)	0.0155 (10)	−0.0007 (10)	0.0095 (9)	0.0029 (9)
N3	0.0180 (11)	0.0158 (12)	0.0224 (11)	−0.0052 (10)	−0.0028 (9)	0.0014 (9)
N4	0.0133 (10)	0.0129 (11)	0.0137 (10)	−0.0021 (9)	−0.0005 (8)	−0.0041 (8)
N5	0.0152 (10)	0.0155 (12)	0.0204 (11)	0.0013 (9)	0.0024 (8)	−0.0062 (9)
N6	0.0107 (10)	0.0170 (12)	0.0218 (11)	−0.0033 (9)	0.0004 (8)	0.0002 (9)
N7	0.0116 (11)	0.0247 (13)	0.0230 (11)	0.0047 (10)	0.0067 (9)	0.0073 (10)
N8	0.0214 (13)	0.0430 (18)	0.0395 (15)	0.0115 (12)	−0.0130 (11)	−0.0242 (13)
N9	0.0182 (11)	0.0278 (14)	0.0164 (10)	0.0039 (10)	0.0095 (9)	0.0017 (9)
N10	0.0244 (12)	0.0274 (14)	0.0120 (10)	0.0092 (10)	0.0041 (9)	0.0011 (9)
O1	0.0232 (10)	0.0269 (12)	0.0175 (9)	0.0033 (9)	0.0002 (8)	−0.0045 (8)
O2	0.0215 (10)	0.0322 (12)	0.0257 (10)	0.0090 (9)	0.0138 (8)	0.0066 (9)
O3	0.0150 (9)	0.0482 (14)	0.0220 (10)	0.0036 (9)	0.0062 (8)	0.0005 (9)
O4	0.0540 (15)	0.0569 (17)	0.0251 (11)	0.0315 (13)	0.0029 (10)	−0.0153 (11)
O5	0.0220 (10)	0.0318 (13)	0.0243 (10)	−0.0029 (9)	0.0009 (8)	0.0046 (9)
C1	0.0132 (12)	0.0152 (14)	0.0250 (13)	−0.0021 (10)	0.0072 (10)	−0.0005 (11)
C2	0.0183 (13)	0.0166 (14)	0.0160 (12)	−0.0004 (11)	0.0032 (10)	−0.0023 (10)
C3	0.0202 (13)	0.0176 (14)	0.0156 (12)	0.0047 (11)	0.0074 (10)	0.0036 (10)
C4	0.0136 (12)	0.0161 (14)	0.0206 (13)	0.0066 (11)	0.0055 (10)	0.0007 (10)
C5	0.0366 (17)	0.0191 (15)	0.0124 (12)	0.0078 (13)	−0.0029 (11)	−0.0016 (11)
C6	0.0273 (15)	0.0253 (16)	0.0162 (12)	0.0102 (13)	0.0088 (11)	0.0034 (11)
C7	0.0118 (12)	0.0224 (15)	0.0210 (13)	0.0002 (11)	0.0046 (10)	0.0069 (11)
C8	0.0121 (12)	0.0182 (14)	0.0163 (12)	0.0056 (10)	0.0019 (9)	0.0049 (10)
C9	0.0174 (13)	0.0157 (14)	0.0177 (12)	0.0039 (11)	−0.0006 (10)	0.0002 (10)
C10	0.0150 (12)	0.0234 (15)	0.0203 (13)	0.0057 (11)	0.0059 (10)	0.0047 (11)
C11	0.0123 (12)	0.0131 (14)	0.0299 (14)	0.0010 (10)	0.0044 (10)	0.0057 (11)
C12	0.0112 (12)	0.0216 (15)	0.0196 (12)	0.0039 (11)	0.0027 (10)	0.0015 (11)
C13	0.0200 (13)	0.0154 (14)	0.0231 (13)	−0.0053 (11)	−0.0014 (11)	0.0016 (11)
C14	0.0221 (14)	0.0160 (14)	0.0226 (13)	0.0025 (12)	0.0102 (11)	−0.0023 (11)
C15	0.0131 (13)	0.0232 (16)	0.0406 (17)	0.0018 (12)	0.0034 (12)	−0.0038 (13)
C16	0.0226 (14)	0.0389 (19)	0.0178 (13)	0.0130 (14)	0.0017 (11)	−0.0045 (13)
C17	0.0234 (14)	0.0221 (16)	0.0148 (12)	0.0022 (12)	0.0026 (10)	0.0006 (11)
C18	0.0175 (13)	0.0200 (15)	0.0223 (13)	0.0027 (11)	0.0039 (10)	0.0049 (11)
C19	0.0208 (14)	0.0291 (17)	0.0181 (13)	0.0077 (12)	0.0078 (11)	0.0071 (12)
C20	0.0223 (14)	0.0182 (15)	0.0142 (12)	0.0035 (12)	0.0051 (10)	0.0064 (10)

### Geometric parameters (Å, °)

**Table:**

Cd1—Cl2	2.5216 (6)	N8—C16	1.378 (4)
Cd1—Cl1	2.5343 (6)	N8—H8A	0.8600
Cd1—Cl5	2.6796 (6)	N9—C17	1.332 (3)
Cd1—Cl3	2.6900 (6)	N9—C20	1.383 (3)
Cd1—Cl4	2.6917 (6)	N9—H9B	0.8600
Cd1—Cl6	2.7670 (6)	N10—C19	1.331 (3)
Cd2—Cl10	2.5184 (6)	N10—C20	1.377 (3)
Cd2—Cl11	2.5273 (6)	N10—H10B	0.8600
Cd2—Cl6	2.6698 (6)	O1—C4	1.212 (3)
Cd2—Cl3	2.6766 (6)	O2—C8	1.212 (3)
Cd2—Cl9	2.7295 (6)	O3—C12	1.209 (3)
Cd2—Cl4	2.7468 (6)	O4—C16	1.209 (3)
Cd3—Cl8	2.5081 (6)	O5—C20	1.205 (3)
Cd3—Cl7	2.5444 (6)	C1—C2	1.363 (3)
Cd3—Cl5	2.6284 (6)	C1—H1B	0.9300
Cd3—Cl9	2.6692 (6)	C2—C3	1.365 (4)
Cd3—Cl4	2.7201 (6)	C2—H2B	0.9300
Cd3—Cl6	2.7214 (6)	C3—H3B	0.9300
N1—C3	1.341 (3)	C5—C6	1.367 (4)
N1—C4	1.377 (3)	C5—H5B	0.9300
N1—H1A	0.8600	C6—C7	1.369 (4)
N2—C1	1.339 (3)	C6—H6B	0.9300
N2—C4	1.386 (3)	C7—H7B	0.9300
N2—H2A	0.8600	C9—C10	1.369 (4)
N3—C5	1.332 (3)	C9—H9A	0.9300
N3—C8	1.376 (3)	C10—C11	1.370 (4)
N3—H3A	0.8600	C10—H10A	0.9300
N4—C7	1.332 (3)	C11—H11A	0.9300
N4—C8	1.377 (3)	C12—O3	1.209 (3)
N4—H4A	0.8600	C13—C14	1.375 (4)
N5—C11	1.338 (3)	C13—H13A	0.9300
N5—C12	1.373 (3)	C14—C15	1.359 (4)
N5—H5A	0.8600	C14—H14A	0.9300
N6—C9	1.339 (3)	C15—H15A	0.9300
N6—C12	1.381 (3)	C17—C18	1.381 (4)
N6—H6A	0.8600	C17—H17A	0.9300
N7—C13	1.333 (3)	C18—C19	1.368 (4)
N7—C16	1.381 (4)	C18—H18A	0.9300
N7—H7A	0.8600	C19—H19A	0.9300
N8—C15	1.325 (4)		
			
Cl2—Cd1—Cl1	98.42 (2)	C13—N7—H7A	117.8
Cl2—Cd1—Cl5	95.56 (2)	C16—N7—H7A	117.8
Cl1—Cd1—Cl5	100.91 (2)	C15—N8—C16	124.9 (2)
Cl2—Cd1—Cl3	97.84 (2)	C15—N8—H8A	117.5
Cl1—Cd1—Cl3	97.61 (2)	C16—N8—H8A	117.5
Cl5—Cd1—Cl3	155.233 (18)	C17—N9—C20	124.3 (2)
Cl2—Cd1—Cl4	167.47 (2)	C17—N9—H9B	117.9
Cl1—Cd1—Cl4	94.048 (19)	C20—N9—H9B	117.9
Cl5—Cd1—Cl4	80.744 (18)	C19—N10—C20	124.4 (2)
Cl3—Cd1—Cl4	81.693 (18)	C19—N10—H10B	117.8
Cl2—Cd1—Cl6	86.881 (19)	C20—N10—H10B	117.8
Cl1—Cd1—Cl6	173.839 (19)	N2—C1—C2	121.1 (2)
Cl5—Cd1—Cl6	81.593 (18)	N2—C1—H1B	119.5
Cl3—Cd1—Cl6	78.455 (18)	C2—C1—H1B	119.5
Cl4—Cd1—Cl6	80.746 (18)	C1—C2—C3	117.2 (2)
Cl10—Cd2—Cl11	93.13 (2)	C1—C2—H2B	121.4
Cl10—Cd2—Cl6	171.98 (2)	C3—C2—H2B	121.4
Cl11—Cd2—Cl6	93.534 (19)	N1—C3—C2	120.6 (2)
Cl10—Cd2—Cl3	103.046 (19)	N1—C3—H3B	119.7
Cl11—Cd2—Cl3	98.080 (19)	C2—C3—H3B	119.7
Cl6—Cd2—Cl3	80.420 (18)	O1—C4—N1	123.3 (2)
Cl10—Cd2—Cl9	93.725 (19)	O1—C4—N2	124.0 (2)
Cl11—Cd2—Cl9	99.466 (19)	N1—C4—N2	112.8 (2)
Cl6—Cd2—Cl9	80.773 (18)	N3—C5—C6	120.6 (2)
Cl3—Cd2—Cl9	154.942 (19)	N3—C5—H5B	119.7
Cl10—Cd2—Cl4	91.835 (19)	C6—C5—H5B	119.7
Cl11—Cd2—Cl4	175.033 (19)	C5—C6—C7	117.0 (2)
Cl6—Cd2—Cl4	81.504 (18)	C5—C6—H6B	121.5
Cl3—Cd2—Cl4	80.921 (18)	C7—C6—H6B	121.5
Cl9—Cd2—Cl4	80.051 (18)	N4—C7—C6	120.4 (2)
Cl8—Cd3—Cl7	95.43 (2)	N4—C7—H7B	119.8
Cl8—Cd3—Cl5	99.23 (2)	C6—C7—H7B	119.8
Cl7—Cd3—Cl5	99.457 (19)	O2—C8—N3	124.9 (2)
Cl8—Cd3—Cl9	95.034 (19)	O2—C8—N4	122.8 (2)
Cl7—Cd3—Cl9	95.419 (19)	N3—C8—N4	112.2 (2)
Cl5—Cd3—Cl9	158.251 (19)	N6—C9—C10	120.7 (2)
Cl8—Cd3—Cl4	168.35 (2)	N6—C9—H9A	119.6
Cl7—Cd3—Cl4	95.99 (2)	C10—C9—H9A	119.6
Cl5—Cd3—Cl4	81.139 (18)	C9—C10—C11	116.8 (2)
Cl9—Cd3—Cl4	81.609 (18)	C9—C10—H10A	121.6
Cl8—Cd3—Cl6	87.40 (2)	C11—C10—H10A	121.6
Cl7—Cd3—Cl6	175.583 (19)	N5—C11—C10	120.8 (2)
Cl5—Cd3—Cl6	83.393 (18)	N5—C11—H11A	119.6
Cl9—Cd3—Cl6	80.932 (18)	C10—C11—H11A	119.6
Cl4—Cd3—Cl6	81.067 (18)	O3—C12—N5	123.8 (2)
Cd2—Cl3—Cd1	84.236 (18)	O3—C12—N5	123.8 (2)
Cd1—Cl4—Cd3	82.031 (17)	O3—C12—N6	123.3 (2)
Cd1—Cl4—Cd2	82.865 (17)	O3—C12—N6	123.3 (2)
Cd3—Cl4—Cd2	81.961 (17)	N5—C12—N6	112.8 (2)
Cd3—Cl5—Cd1	83.991 (17)	N7—C13—C14	120.6 (2)
Cd2—Cl6—Cd3	83.364 (17)	N7—C13—H13A	119.7
Cd2—Cl6—Cd1	82.884 (17)	C14—C13—H13A	119.7
Cd3—Cl6—Cd1	80.645 (17)	C15—C14—C13	116.7 (3)
Cd3—Cl9—Cd2	83.220 (16)	C15—C14—H14A	121.6
C3—N1—C4	124.5 (2)	C13—C14—H14A	121.6
C3—N1—H1A	117.8	N8—C15—C14	120.9 (3)
C4—N1—H1A	117.8	N8—C15—H15A	119.6
C1—N2—C4	123.9 (2)	C14—C15—H15A	119.6
C1—N2—H2A	118.0	O4—C16—N8	124.1 (3)
C4—N2—H2A	118.0	O4—C16—N7	123.8 (3)
C5—N3—C8	124.8 (2)	N8—C16—N7	112.1 (2)
C5—N3—H3A	117.6	N9—C17—C18	120.4 (2)
C8—N3—H3A	117.6	N9—C17—H17A	119.8
C7—N4—C8	125.0 (2)	C18—C17—H17A	119.8
C7—N4—H4A	117.5	C19—C18—C17	117.0 (2)
C8—N4—H4A	117.5	C19—C18—H18A	121.5
C11—N5—C12	124.5 (2)	C17—C18—H18A	121.5
C11—N5—H5A	117.8	N10—C19—C18	120.8 (2)
C12—N5—H5A	117.8	N10—C19—H19A	119.6
C9—N6—C12	124.3 (2)	C18—C19—H19A	119.6
C9—N6—H6A	117.8	O5—C20—N10	123.4 (2)
C12—N6—H6A	117.8	O5—C20—N9	123.6 (2)
C13—N7—C16	124.3 (2)	N10—C20—N9	113.0 (2)
			
Cl10—Cd2—Cl3—Cd1	−129.506 (19)	Cl4—Cd3—Cl6—Cd1	−42.320 (15)
Cl11—Cd2—Cl3—Cd1	135.360 (19)	Cl2—Cd1—Cl6—Cd2	140.506 (19)
Cl6—Cd2—Cl3—Cd1	43.114 (17)	Cl5—Cd1—Cl6—Cd2	−123.400 (18)
Cl9—Cd2—Cl3—Cd1	1.27 (5)	Cl3—Cd1—Cl6—Cd2	41.845 (17)
Cl4—Cd2—Cl3—Cd1	−39.720 (17)	Cl4—Cd1—Cl6—Cd2	−41.486 (17)
Cl2—Cd1—Cl3—Cd2	−126.750 (19)	Cl2—Cd1—Cl6—Cd3	−135.087 (19)
Cl1—Cd1—Cl3—Cd2	133.608 (19)	Cl5—Cd1—Cl6—Cd3	−38.993 (16)
Cl5—Cd1—Cl3—Cd2	−4.61 (5)	Cl3—Cd1—Cl6—Cd3	126.253 (18)
Cl4—Cd1—Cl3—Cd2	40.601 (17)	Cl4—Cd1—Cl6—Cd3	42.921 (16)
Cl6—Cd1—Cl3—Cd2	−41.579 (16)	Cl8—Cd3—Cl9—Cd2	126.796 (19)
Cl2—Cd1—Cl4—Cd3	−33.55 (10)	Cl7—Cd3—Cl9—Cd2	−137.247 (19)
Cl1—Cd1—Cl4—Cd3	140.568 (18)	Cl5—Cd3—Cl9—Cd2	−4.15 (6)
Cl5—Cd1—Cl4—Cd3	40.154 (16)	Cl4—Cd3—Cl9—Cd2	−41.973 (17)
Cl3—Cd1—Cl4—Cd3	−122.314 (18)	Cl6—Cd3—Cl9—Cd2	40.245 (17)
Cl6—Cd1—Cl4—Cd3	−42.750 (16)	Cl10—Cd2—Cl9—Cd3	132.909 (19)
Cl2—Cd1—Cl4—Cd2	49.29 (10)	Cl11—Cd2—Cl9—Cd3	−133.288 (18)
Cl1—Cd1—Cl4—Cd2	−136.601 (18)	Cl6—Cd2—Cl9—Cd3	−41.211 (17)
Cl5—Cd1—Cl4—Cd2	122.985 (18)	Cl3—Cd2—Cl9—Cd3	0.58 (5)
Cl3—Cd1—Cl4—Cd2	−39.483 (17)	Cl4—Cd2—Cl9—Cd3	41.697 (17)
Cl6—Cd1—Cl4—Cd2	40.081 (16)	C4—N2—C1—C2	−0.5 (4)
Cl8—Cd3—Cl4—Cd1	51.69 (10)	N2—C1—C2—C3	1.0 (4)
Cl7—Cd3—Cl4—Cd1	−139.723 (17)	C4—N1—C3—C2	1.4 (4)
Cl5—Cd3—Cl4—Cd1	−41.047 (17)	C1—C2—C3—N1	−1.5 (4)
Cl9—Cd3—Cl4—Cd1	125.659 (19)	C3—N1—C4—O1	−180.0 (2)
Cl6—Cd3—Cl4—Cd1	43.596 (16)	C3—N1—C4—N2	−0.8 (3)
Cl8—Cd3—Cl4—Cd2	−32.17 (10)	C1—N2—C4—O1	179.5 (2)
Cl7—Cd3—Cl4—Cd2	136.413 (17)	C1—N2—C4—N1	0.3 (3)
Cl5—Cd3—Cl4—Cd2	−124.911 (18)	C8—N3—C5—C6	0.2 (4)
Cl9—Cd3—Cl4—Cd2	41.795 (16)	N3—C5—C6—C7	0.8 (4)
Cl6—Cd3—Cl4—Cd2	−40.269 (15)	C8—N4—C7—C6	−0.4 (4)
Cl10—Cd2—Cl4—Cd1	142.739 (18)	C5—C6—C7—N4	−0.7 (4)
Cl6—Cd2—Cl4—Cd1	−41.755 (16)	C5—N3—C8—O2	177.7 (2)
Cl3—Cd2—Cl4—Cd1	39.818 (17)	C5—N3—C8—N4	−1.2 (3)
Cl9—Cd2—Cl4—Cd1	−123.800 (18)	C7—N4—C8—O2	−177.7 (2)
Cl10—Cd2—Cl4—Cd3	−134.350 (18)	C7—N4—C8—N3	1.3 (3)
Cl6—Cd2—Cl4—Cd3	41.155 (16)	C12—N6—C9—C10	−1.5 (4)
Cl3—Cd2—Cl4—Cd3	122.729 (18)	N6—C9—C10—C11	1.1 (4)
Cl9—Cd2—Cl4—Cd3	−40.889 (16)	C12—N5—C11—C10	1.2 (4)
Cl8—Cd3—Cl5—Cd1	−127.150 (19)	C9—C10—C11—N5	−1.0 (4)
Cl7—Cd3—Cl5—Cd1	135.712 (19)	O3—O3—C12—N5	0.0 (7)
Cl9—Cd3—Cl5—Cd1	3.19 (6)	O3—O3—C12—N6	0.0 (6)
Cl4—Cd3—Cl5—Cd1	41.062 (17)	C11—N5—C12—O3	178.5 (2)
Cl6—Cd3—Cl5—Cd1	−40.881 (17)	C11—N5—C12—O3	178.5 (2)
Cl2—Cd1—Cl5—Cd3	126.274 (19)	C11—N5—C12—N6	−1.4 (3)
Cl1—Cd1—Cl5—Cd3	−134.022 (19)	C9—N6—C12—O3	−178.3 (3)
Cl3—Cd1—Cl5—Cd3	3.71 (5)	C9—N6—C12—O3	−178.3 (3)
Cl4—Cd1—Cl5—Cd3	−41.648 (17)	C9—N6—C12—N5	1.5 (3)
Cl6—Cd1—Cl5—Cd3	40.268 (17)	C16—N7—C13—C14	3.4 (4)
Cl11—Cd2—Cl6—Cd3	139.268 (17)	N7—C13—C14—C15	3.4 (4)
Cl3—Cd2—Cl6—Cd3	−123.124 (18)	C16—N8—C15—C14	1.5 (5)
Cl9—Cd2—Cl6—Cd3	40.241 (16)	C13—C14—C15—N8	−5.8 (4)
Cl4—Cd2—Cl6—Cd3	−40.974 (16)	C15—N8—C16—O4	−175.5 (3)
Cl11—Cd2—Cl6—Cd1	−139.382 (17)	C15—N8—C16—N7	4.8 (5)
Cl3—Cd2—Cl6—Cd1	−41.774 (17)	C13—N7—C16—O4	173.0 (3)
Cl9—Cd2—Cl6—Cd1	121.591 (18)	C13—N7—C16—N8	−7.2 (4)
Cl4—Cd2—Cl6—Cd1	40.375 (16)	C20—N9—C17—C18	−2.7 (4)
Cl8—Cd3—Cl6—Cd2	−136.844 (18)	N9—C17—C18—C19	0.0 (4)
Cl5—Cd3—Cl6—Cd2	123.549 (18)	C20—N10—C19—C18	0.6 (4)
Cl9—Cd3—Cl6—Cd2	−41.324 (17)	C17—C18—C19—N10	1.0 (4)
Cl4—Cd3—Cl6—Cd2	41.525 (16)	C19—N10—C20—O5	177.1 (3)
Cl8—Cd3—Cl6—Cd1	139.312 (18)	C19—N10—C20—N9	−3.0 (4)
Cl5—Cd3—Cl6—Cd1	39.704 (17)	C17—N9—C20—O5	−176.0 (3)
Cl9—Cd3—Cl6—Cd1	−125.168 (18)	C17—N9—C20—N10	4.1 (4)

### Hydrogen-bond geometry (Å, °)

**Table:**

*D*—H···*A*	*D*—H	H···*A*	*D*···*A*	*D*—H···*A*
N2—H2A···Cl11^i^	0.86	2.56	3.246 (2)	138
N3—H3A···Cl8^ii^	0.86	2.37	3.104 (2)	144
N4—H4A···Cl10^iii^	0.86	2.31	3.160 (2)	169
N5—H5A···Cl7^iv^	0.86	2.41	3.194 (2)	151
N6—H6A···Cl1^v^	0.86	2.31	3.138 (2)	162
N7—H7A···O2^vi^	0.86	2.03	2.880 (3)	167
N10—H10B···Cl7^vi^	0.86	2.54	3.349 (2)	157
N8—H8A···O5^vii^	0.86	2.28	2.804 (3)	120
N8—H8A···O4^viii^	0.86	2.34	3.117 (4)	150
N9—H9B···O3	0.86	2.13	2.920 (3)	152

Symmetry codes: (i) −*x*, *y*+1/2, −*z*+1/2; (ii) *x*, *y*+1, *z*; (iii) −*x*, −*y*+1, −*z*; (iv) −*x*+1, −*y*+1, −*z*+1; (v) *x*, −*y*+1/2, *z*+1/2; (vi) *x*, *y*, *z*+1; (vii) −*x*+1, −*y*+1, −*z*+2; (viii) −*x*+1, −*y*+2, −*z*+2.
